# Premature Ejaculation: From Physiology to Treatment 

**Published:** 2019-09

**Authors:** Mário Pereira-Lourenço, Duarte Vieira e Brito, Bruno Jorge Pereira

**Affiliations:** 1 Urology Department, Francisco Gentil Portuguese Institute of Oncology, Coimbra, Portugal; 2 Urology Department, Cova da Beira University Hospital Centre, Covilhã, Portugal

**Keywords:** Premature Ejaculation, Physiology, Treatment, Sexual Dysfunction

## Abstract

**Objective:** To review in literature about the concept of premature ejaculation from physiology to treatment.

**Materials and methods:** A literature search conducted with Pubmed and Cochrane.

**Results:** An accurate clinical history is the best diagnostic method, and in the majority of the cases it is enough to differentiate between primary and acquired premature ejaculation. Nowadays the treatment is not curative but is effective in increasing the *Intravaginal Ejaculatory Latency Time*, improving the couple’s sexual satisfaction.

**Conclusion:** Although PE is the most frequent sexual dysfunction, it is still sub-diagnosed. Combining behavioural techniques with pharmacotherapy is the best way of treatment.

## Introduction

Premature ejaculation (PE) is the most prevalent sexual dysfunction in men. Historical ambiguity around PE is due to difficulty in conducting quality research when there is not a universal definition for this disease. The perception of normal ejaculatory latency time is subjective and has strong cultural and socioeconomical influences (1).

The development of Sexual Medicine led to a much better understanding of PE, no longer being considered a purely psychological disorder and being understood in a physiopathological basis. This evolution led to the development of new ways of treatment (2).

## Materials and methods

***Information sources:*** A literature search was conducted with Pubmed and Cochrane. Supplementary literature searches included examining the reference lists of all relevant studies, pertinent review articles, and meta-analyses. The European Society of Sexual Medicine manual was included in this review.

***Anatomy and Physiology of Ejaculation: ***Male sexual response is described as a succession of intimately related phases: desire, arousal, orgasmand resolution. Ejaculation is the culmination of the male sexual cycle, although distinct from orgasm (3).

Ejaculatory response consists in the succession of two events: emission and expulsion. Emission consists in the secretion of spermatozoa and components of seminal liquid into the prostatic urethra. Once concluded, expulsion begins in the form of vigorous rhythmic contractions from the pelviperenealstriated muscles and striated urethral sphincter, allowing propulsion of semen. Expulsion results from the maximal activation of the sympathetic nervous system resulting in rhythmic spasm of perineal muscles, and coordination between the urethral internal and external sphincters. 

**Table 1:** Male genital organs involved in the process of ejaculation

	**Organ**	**Physiology/Function**
Emission	Epididymis	Spermatozoa maturation Storage
	*Vas deferens*	Peristalsis, transport of spermatozoa to prostatic urethra (by the *ductus * *deferens*)
	Seminal Vesicles	Pair of tubular glands with stroma composed of smooth muscle, whose epithelial cells produce 50-80% of the ejaculatory volume
	Prostate	Produces 15-30% of seminal fluid
	Bulbourethral or Cowper glands	Enveloped by a layer of straited muscle. Minimal production of fluid
Expulsion	Bladder neck	Smooth muscle cells Contraction during expulsion prevents backward flow into the bladder
	Urethra	Stratified epithelium, surrounded in more than half its length by muscle fibers that form the external urethral sphincter (presents strong contractions interrupted by periods of “silence” during expulsion)
	Pelvipereneal striated muscles	Includes the levatorani, ischiocavernosus and bulbospongiosus (with the latest having a preponderant role in propelling the semen through rhythmic contractions).

*Adapted from Clement et al. (3)

These rhythmic contractions are extremely pleasurable and are part of the sensation of orgasm. A young heathy individual has 10 to 15 contractions per ejaculation, as frequency reduces during orgasm (3, 4).

***Anatomy: ***Male genital anatomy involved in the emission process is different from the one involved in the expulsion process.The anatomical structures that participate in the emission and expulsion are summarized in table 1.

***Peripheral nervous system:*** Activation of the autonomous nervous system and the somatic nervous system is essential for ejaculation [as is the role of non-adrenergic non-cholinergic (NANC) in modulating the activity of accessory glands] (3). 

Neuro-anatomical studies indicate that the parasympathetic is responsible for epithelial secretion, while the sympathetic nervous system is responsible for tonal control and contractions of smooth muscle in the seminal trait (3).

Clinical observation confirmed the fundamental role of the sympathetic nervous system in the urogenital control in men. Anejaculation or retrograde ejaculation are consequences described in patients with efferent sympathetic nerve lesions.Electrical stimulation of the hypogastric plexus, which contains post-ganglionic sympathetic neurons, allows for the collection of semen in paraplegic men (3).

Anterograde ejaculatory flow depends of the perfect synchronization of thestriated pelvipereneal muscles and external urethral sphincter. The pudendal nerve is responsible for the motor enervation of these muscles (3).

Genital sensory afferents enervation also has a crucial role in ejaculation, being different contractile responses observed with different sensitive stimuli. For example, penile vibratory stimulation applied to the gland of men with spinal cord injuries allows for the collection of sperm in more than 50% of cases (5).

***Spinal control: ***Different spinal areas control ejaculation. Pre-ganglionic sympathetic neurons located in the intermediolateral cell column and the dorsal grey column (T12- L2) innervate the urogenital area. Pre-ganglionic parasympathetic that innervate the urogenital area are found in intermediolateral cell column between S2-S4. Somatic neurons responsible for motor enervation of pelvipereneal and urethral sphincter are found on the Onuf nucleus on the ventral horn of S2-S4. A group of spinal interneurons have been identified in animal models as ejaculation generators (3). A key component to the generation of ejaculation was identified on the lumbar region of L3-L4. These spinothalamic neuros segments, also known as spinothalamic ejaculation generators, coordinate the autonomous and somatic (pudendal) processes, in order to initiate ejaculation. These interneurons are part of and coordinate somatosensory information, as the supraspinal excitatory and inhibitory impulses (3, 4).

***Cerebral control:*** Cerebral control of ejaculation is complex and results of interaction between various neural groups located in different cerebral areas, divided in sensitive/integrative, excitatory and inhibitory. The cerebral areas that actively participate in ejaculation are the posteromedial division of the bed nucleus of the striaterminals (BNSTpm), the posterodorsal area of the amygdala (MeApd), the posterodorsal pre-optic nucleus (PNpd)and the parvicellular part of the subparafascicular thalamus (SPFp). Additionally, subparafascicular thalamus receives direct enervation from spinothalamic neurons (3, 4).

One of the most important excitatory paths includes neurons in the pre-optic medial area (MPOA)that project in the paraventricular hypothalamus (PVN), whose axons communicate with autonomic neurons in the medullar ejaculation centers (3, 4).

The inhibitorycentres originate from neurons in the gigantocellular nuclei (Gi) and ventral raphe in the ventral medulla.Imaging studies show intense neural activity in the mesodiencephalic, thalamus (medial, ventral and subparafascilar areas) and cerebral cortex at ejaculation (4).

**Figure 1:**
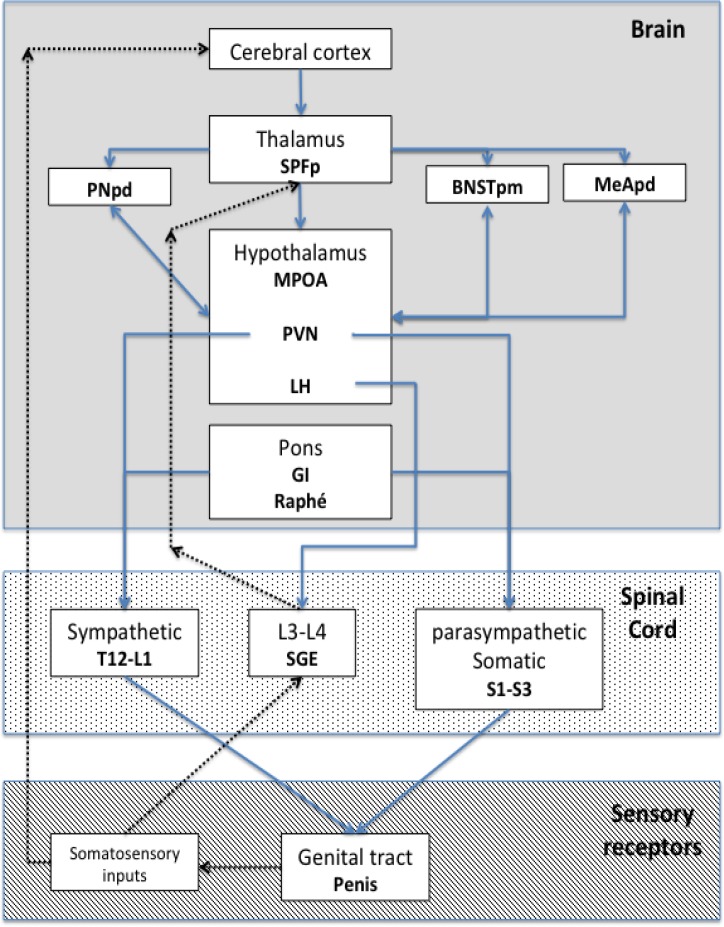
Central control of ejaculation and connections with medullar and peripheric control. Adapted from Clemet et al.    (3)

The complex system between cerebral cortex, spinal cord and sensory receptors is summarized in figure 1.

***Targets and neurotransmitters involved in ejaculation:*** Neurotransmitters involved in the peripheral control, medullar and central of ejaculationare summarized in table 2. 

***Definition of premature ejaculation:*** The first medical definition of PE dates from 1970 by Masters & Johnson, defining EP as “*the inability of a man to delay ejaculation long enough for his partner to reach orgasm on 50% of intercourse attempts”*(1). In the Eighties the American Psychiatry Association in *Diagnostic and Statistical Manual of Mental Disorders 3rd Edition* (DSM-III), defined PE as the inability to voluntarily control ejaculation. The next version (DSM-IV) eliminated the term “ejaculation control”, giving emphasis to “short time” to ejaculation. In 1994, the*International Statistical Classification of Disease 10th Edition* (ICD-10) introduced in the definition of PE the term *Intravaginal Ejaculatory Latency Time (*IELT), being an IELT inferior to 15 considered PE, although scientific support for this *cut-off *was not clear (1, 4).

BNSTpm: posteromedial division of the bed nucleus of the stria terminals; Gi:gigantocellular nuclei; LH: lateral hypothalamus; MeApd:the posterodorsal area of the amygdala; MPOA :pre-optic medial area; PNpd:posterodorsal pre-optic nucleus; PVN:paraventricular hypothalamus; SGE: ejaculatory spinal generator; SPFp:subparafascicular thalamus.

In 2007 the International* Society for Sexual Medicine* (ISSM) defined PE using 3 principles: 1) reduced IELT; 2) perception of lack of ejaculatory control; 3) negative personal consequences such as stress, frustration, sexual avoidanceor difficulty in interpersonal relationships (1, 6).

The most recent definitions are presented in table 3. 

***Classification:*** Schapiro subdivided PE in hypotonic (associated with erectile dysfunction) and hypertonic (7). Others divisions of PE in psychogenic and biogenic were proposed, without great acceptance (1). Currently, the most accepted andused classification consists in the division of PE in primary (*lifelong*) and secondary (acquired). Primary PE occurs since the first sexual relation, while secondary PE happens in an individual who has experienced “normal” sexual relations.

**Table 2:** Neurotransmitters involved in the ejaculatory process at peripheral, medullar, central levels*

	**Neurotransmitter**	**Function/Comment**
Peripheral control	Norepinephrine	Contraction of seminal vesicles; α-1 receptors in smooth muscle.
	Acetylcholine	Contraction of seminal vesicles; Muscarinic receptor.
	Nitric oxide	Major component of NANC autonomic system; Contraction of seminal vesicles.
	Oxytocin	Produced mostly in the pituitary glands; Contraction of seminal apparatus.
	Purines	ATP dependent *vas deferens* contraction.
	Serotonin	Facilitates expulsion reflex (5HT2); Limited role.
	Sensitive receptors	Well demonstrated function by the effect of topical anaesthesia.
Medullar control	GABA	Scattered presence in the no CNS; Subarachnoid application of baclofen (GABA antagonist) prevents ejaculation.
	Oxytocin	Produced in the parvocellular neurons stored in the hypothalamus; Oxytocinergic terminals have been identified in the intermediolateral (such as the sacral parasympathetic nucleus); Modulates autonomic centers, inhibits ejaculation (antagonistic effect of its central action).
		Various subtypes of receptors in different locations (pre/post-synapti, auto/heteroreceptors), with different functions (excitatory/inhibitory); Characterization is difficult.
	P substance	Involved in sensory transmission; NK1 receptor found mostly in lumbar spinothalamic neurons; NK1 antagonists cause anejaculation.
Central	Serotonin	SRIs historically associated with delayed ejaculation (extracellular 5- HT increases ejaculating time); Different receptors with different actions (5HT1A promote ejaculation, 5HT1B inhibits ejaculation, 5-HT2A/C inhibits ejaculation).
	Dopamine	Acts mostly on the hypothalamus Pro-ejaculatory action; Essential in the paraventricular nucleus and medial pre-optic areas; D2-type receptors (D2 antagonists used in treatment of schizophrenia ejaculation) prevent ejaculation.
	Opioids	Endogenous opioids have inhibitory effects.
	Oxytocin	Stimulating effect.

ATP: adenosine triphosphate; GABA: gamma-aminobutyric acid; NANC: Non adrenergic noncholinergic enervation; NK1: Neurokinin 1; CNS: central nervous system; SRIs Serotonin reuptake inhibitors; 5HT:5-hydroxytryptamine receptors.

* Adapted from Clement et al. (3)

Primary PE present the following characteristics : 1) since the first sexual intercourse; 2) present in all (or almost all) partners, in more than 80-90% of sexual intercourses; 3)without significant change in IELT with age, although 25-30% present worsening at around 30-35 years of age ; 4) more than 85% of men ejaculate in the first minute, while 10-20% ejaculate between the first and second minute; 5) symptoms cause distress (8).

Secondary PE, appears in a set moment of life and it assumes a superior IELTin relation to primary PE (until 3 minutes as defined by ISSM) (6).

Some authors, observing discrepancies between IELT inferior to 1 minute (~2,5%) and the incidence (superior)of subjective/self-reported PE in men with IELT superior to 1 minute, added two more subtypes to PE: variable PE and subjective PE (table 4) (1).

**Table 3:** Recent definitions for Premature Ejaculation

**Author**	**Definition**
*International Society for Sexual Medicine* (ISSM), 2014 (6)	- Ejaculation that occurs always or almost always within the first minute after vaginal penetration since the first sexual intercourse (primary PE) or a clinical significative reduction in IELT, usually 3 minutes after penetration (secondary PE). - Inability to slow ejaculation in all or almost all vaginal intercourses. - Negative personal consequences such as stress, frustration or sexual avoidance.
*Diagnostic and Statistical Manual of * *Mental Disorders 5* ^th^ * Edition* (DSM-V), 2013 (55)	- Persistent and recurrent ejaculatory pattern of IELT of 1 minute or less after vaginal penetration or before the desire of the individual. - Duration of more than 6 months. - Causes clinically relevant stress.
*International Statistical Classification of * *Diseases and Related Health Problems 10* ^th^ *Revision* (ICD-10), 2016 (56)	- Inability to control ejaculation in such a way that both elements of the couple feel sexual satisfied.

PE: premature ejaculation; IELT: Intravaginal Ejaculatory Latency Time.

***Etiology and risk factors:*** Risk factorsdiffer between primary and secondary PE.

In relation to primary PE the following factors/hypotheses are presented:

- Genetic and neurobiological hypothesis (8)

- Possible relation with low central serotonin levels.
- Does not present a Mendelian hereditary pattern.

- Genetic polimorfisms (8)

- Janssen et al.demonstrated a very important role of the 5-HTTLPR polymorphism in IELT. In 89 patients with this polymorphism, 83 ejaculated in less than a minute (9).
- The same authors studied the C(1019)G polymorphism of the 5HT1A receptor and the Cys23Ser polymorphism of the 5HT1C receptor , detecting significant differences in IELTin patients with different genotypes (10, 11).

In relation to secondary PE the following risk factors/etiologies where identified: 

- Psychorelational (12)

- Well-established relation between anxiety, depression and PE.

-Sexual dysfunction (12)

- Relation between erectile dysfunction (ED), hypoactive sexual desire, partner sexual dysfunction and PE.

- Endocrine (6)

- Low levels of testosterone: testosterone facilitates control of ejaculatory reflex.
- Low prolactin and hyperthyroidism are associated with PE.

- Urologic (12)

- Chronic prostatitis and chronic pelvic pain syndrome are associated with PE. Prevalence of PE in patients with chronic prostatitis is around 26% to 77%.
- Circumcision presents conflicting results. Population studies do not show significant difference in IELT between circumcised and uncircumcised men (13). Additionally, there were no differences in sexual function in men assessed before and after circumcision (14). There are although other studies that show that circumcision can increase ejaculatory control (15).

- Idiopathic (12)

***Epidemiology:*** Before determining true epidemiology of PE as a clinical entity, it is important to define “normal” IELT and the sociocultural factors that influence it.

**Table 4:** Classification of Premature Ejaculation*

**CLASSIFICATION**	**Possible ethology**	**Prevalence** * ** (%)**
Primary PE	Functional biological disturbance	2.3 to 3.2
Secondary PE	Phycological, medical or interpersonal	3.9 to 4.8
Variable PE	Normal variant	8.5 to 11.4
Subjective PE	Cultural or abnormal phycological constructions	5.1 to 6.4

* Prevalence based on studies of Turkish and Chinese populations (57, 58). Adapted from Parnham et al. (1)

Waldinger et al. studied IELT (measured with a blinded timer device) in 474 men with normal sexual function from five countries (Holland, United Kingdom, Spain, Turkey and United States of America). Mean and median IELT where 5.7 minutes and 6.0 minutes, respectively (13). Another previous population study by Waldinger et al. in 491 men from the same five countries using stopwatch technique identified a median IELT of 5.4 minutes (0.55-44.1 min), with 0.5% and 2.5% of men presenting an IELT inferior to 0.9 minutesand1.3 minutes, respectively) (16, 17).

There is an overlap in IELT between men with and without PE. This overlap was demonstrated in a populational study from Patrick et al., who compared IELT from 1,380 men without PE and 207 men with PE (defined by DSM-IV). Although IELT is useful in differentiating between the two groups (median IELT of 7.3 minutes in men without PE, comparing with a median IELT of 1.8 minutes in men with PE), there is an overlap between them. In the previous study, 6% of men without PE ejaculated in the first 2 minutes after penetration and 30% ejaculate between the first 2 to 5 minutes (18). 

True epidemiology of PE depends on the tools used to define PE. In one online study based on questionnaires (that did not define a limit to IELT), more than 12,000 men participated from the United States of America, Germany and Italy, showed a prevalence of 22.7% for PE, similar between the three countries. In this study, men with PE referred a higher prevalence of other sexual dysfunctions (anorgasmia, reduced libido, erectile dysfunction) and other psychological disturbances (depression, anxiety, excessive stress). 

Prevalence of PE is relatively stable between different age groups until the seventh decade of life (19-21). There are social, ethnic and cultural factors that influence the epidemiology of PE. A study of more than13,000 men by Laumann et al., observed a reduced prevalence of PE in Islamic countries. According to the authors, this low prevalence in Islamic countries can be explained by cultural perception that a fast ejaculation is a sign of masculinity (20, 21).

A review on the epidemiology of PE showed great difficulty in reaching a universal value, as the methods used to diagnose are very distinct. That review studied 38 publish papers from 1998 to 2004, prevalence of PE varied from 3% (PE defined as IELT< 1 minute, randomized study) to 83.7% (PE defined as occasional occurrence of involuntary ejaculation, online study) (22). 

***Quality of life: ***PE is associated with higher levels of personal stress, in men and partner (4, 23).

Men with PE show higher difficulty in maintaining a relationship, low self-esteem, low self-confidence and higher anxiety rates related to sexual activity (4, 23, 24). 

The severity of PE and its effects in the couple sexuality, is understood by the men as graver when compared with the partner (25).To women, the main problem with PE resides in the focus given by the man to its own sexual performance, resulting in a lack of attention to the partner (26). The negative impact of PE in women is not negligible; it can be the reason to end a relationship (a study showed that almost 25% of women ended a relationship due to their partners PE (27).

Patient evaluation

***Anamnesis: ***There are multiple questionnaires that aid in the diagnosis of PE, the most relevant (for western populations) being Pr*emature Ejaculation Diagnostic Tool *(PEDT) and *Premature Ejaculation Profile *(PEP) (28, 29).

The application of questionnaires can help diagnose and assess the severity of PE, although they are mainly used in clinical research. Concerning clinical practice, the International* Society for Sexual Medicine*, recommends that the following questions be asked (30):

- What is the time between penetration to ejaculation?
- Can you delay ejaculation?
- Do you feel bothered, annoyed or frustrated with your premature ejaculation?

Other aspects must be investigated, such as the existence of erectile dysfunction, impact of the disease in the couple’s relationship, previous treatments and change in quality of life.

In cases of primary PE, it is essential to exclude psychological/psychiatric problems, as these have increased prevalence in these populations. In secondary PE, it is important to characterize previous sexual relations (where PE didn´t exist), describe the current relationship from a sexual and nonsexual point of view, the presence of erectile dysfunction and coitus capacity, the context of when PE occurs and the partners sexual response.

***Physical exam:*** Physical exam is usually normal, namely in patients with primary PE.

However, patients with secondary PE, may have an altered physical exam that suggest a certain aetiology such as the presence of phimoses or frenulum breve, signs of venereal disease, prostatitis or hypogonadism. Summary Neurological examination can be useful.

***Complementary exams:*** Exams should be selected based on suspected aetiology. It is advised to perform, blood count, lipid profile, glycated Haemoglobin, urinalysis, testosterone (free and total), prolactin and Thyroid Stimulating Hormone (TSH) (4, 31).

***Behavioral Therapy:*** First described in 1956 by James H. Semans, the “*stop and start*” technique aims to obtain higher ejaculatory control through immediate coitus or masturbation suspension, returning when there is decreased excitatory stimuli (32). Later, William Masters and Virginia Johnson (1970) perfected the “*stop and start*” technique using the “*squeeze*” method which consist in the application of firm pressure in the gland results in reduced sensibility of that area (33). The authors claimed success rates of 95% (34).

There are other behavioural techniques such as sensory abstraction and thought redirection, pre-coitus masturbation and use of climax control condoms. Cognitive or sexual therapy focuses on perception and feelings with the goal of improving communication between partners, increasing sexual skills, self-confidence and reduce anxiety during sexual intercourse (35). It is believed that behavioural therapies result in increased IELT (some authors claim an increase of 8 times), but there isn´t concrete data to support this claim (36-38). Short term improvement was observed using behavioural methodologies, however there is limited data on the effectiveness of these procedures on the long term (39). Two recently publish metanalyses concluded that there is inconsistent evidence on the effectiveness of psychological intervention in treating PE, confirming the need for future research (40, 41). Regardless, there is a general consensus that combining behavioural techniques with pharmacotherapy can be superior to monotherapy and provide better results on the long term (39).

***Disease-targeted treatment*** ***:*** Organic dysfunctions can be the genesis of secondary premature ejaculation. In these patients, depending on aetiology, it is recommended physical activity, weight loss, abstaining from alcohol, treatment of hyperthyroidism and hypogonadism, strict glycaemic control in diabetic patients and antibiotics in patients with chronic bacterial prostitis (39). It is estimated that 50% of individuals with hyperthyroidism present with premature ejaculation. This prevalence diminishes to 15% when treatment for thyroid disease is initiated (39). Totally 26 to 77% of men with chronic prostatitis or chronic pelvic pain syndrome report PE. As such, antibiotic treatment of chronic bacterial prostatitis seems to have therapeutic benefits in ejaculatory dysfunction (39).

Pharmacological treatment

***Topical anaesthesia:*** The combination of lidocaine and prilocaine topical anaesthetics are the most studied for the treatment of PE (42, 43). One randomized trial demonstrated that applying a creme with 5%lidocaine-prilocaine was effective in prolonging mean IELT in6 to 8 minutes (while the placebo group showed a 1 to 2 minutes increase in mean) (42, 43). Recently a eutectic formula of lidocaine (150 mg/mL) and prilocaine (50 mg/mL) emerged. This cutaneous spray solution applied to the gland is rapidly absorbed in non-keratinized epithelium and has effect 5 minutes right after application. It is odourless, doesn´t require the use of a condom as it creates a thin layer adherent to the application site. Clinical trials demonstrated consistent efficiency in practically all patients (88%) and a median increase in basal IELT of 6 times, improving ejaculatory control and couple´s satisfaction. Local side effects are minimal, generally well tolerated, temporary and are limited to hypoesthesia, loss of erection, genital erythema and local burn (44). According to the *International Society for Sexual Medicine *(ISSM) guidelines “on-demand” topical treatment is well established in treating premature ejaculation (evidence level 1a) (30, 39).

***Dapoxetine:*** Serotonin is the most relevant neurotransmitter in ejaculatory control (34). Dapoxetine is an Serotonin Selective Reuptake Inhibitor (SSRI) with fast onset of action and short duration (maximum blood levels reached after one hour and half and clearance of 95% ate 24 hours) comparing to other SSRIs (Table 5) (45). It is available in two distinct dosages, 30mg and 60 mg, on demand usage, 1 to 3 hours before sexual relations. Its efficiency is likely similar in cases of primary PE and acquired PE and it is predicted a 2.5 to 3increase in mean IELT (30, 34, 39). It should not be administered in patients taking other CYP3A4 inhibitors or other SSRIs and tricyclic antidepressant (TCAs). It is contraindicated in cases of moderate to severe hepatic disease, heart failure, ischemic cardiac disease and carriers of pacemakers. 

**Table 5:** Potential future therapeutics

***CENTRAL *** **TARGETS– Potential treatment options**	
EPELSIBAN	Although oral epelsiban did not present the expected results, intrathecal administration or intracerebroventricular administration may be more effective.
DOPAMINE ANTAGONISTS	Dopamine antagonists may be promising alternatives in treating PE as catecholamines stimulate ejaculation.
DA-8031	5-HT transporter is an important target, as it has the pharmacokinetic profile of a strong SSRI, but with low affinity for other receptors reducing side effects. DA- 8031 has been widely studied in animals with largely positive results.
WAY-100635	5-HT1A/1B receptor antagonists may increase ejaculatory time and diminish anxiety in men with or without PE.
FOLIC ACID AND *SATUREJA * *MONTANA*	The hypotheses of supplementing with folic acid and *Saturejamontana*may be acceptable alternatives to conventional therapies. Although further research is needed as current results are scarce.
**PERIPHERALTARGETS– Potential treatment options**	
SILODOSIN	Seems to be a promising drug, with a good safety profile and favourable global patient satisfaction.
RHO-KINASE INHIBITORS	Speculative target. No clinical experience. Wide distribution through the human body.
ANTAGONISTS OF PURINERGIC RECEPTORS	The role of this receptors remains unknown and more studies are required.
BOTULINUM-A TOXIN	According to animal trials, it may be a promising treatment option.
RESINIFERATOXIN	Good results, corresponding to total sexual satisfaction, where found. Tolerable side effects and robust IELTS where found making it a potential future treatment, especially in patients with redundant foreskin.

* Adapted from Simões-Paço et al. (53)

Side effects are usually well tolerated, and as such, it has limited discontinuation rates (4% for dosage of 30 mg and 10% for dosage of 60 mg). In both dosages the incidence of side effects was 12% which include nauseas, headache, dizziness, diarrhoea, in accordance to the largest observational study (*PAUSE Study*) (46). For the reasons presented, the *International Society for Sexual Medicine *recommends its use on*-demand (*evidence level 1a) (30, 39).

***Other Selective Serotonin Reuptake Inhibitors (SSRIs):*** SSRIs are used off-label as one of the side effects of this class is delayed ejaculation. In a prospective study with 344 patients under treatment with SSRIs for psychiatric illness, a delay in ejaculatory time was found in 46 to 59% of cases (47). In other similar study, with 1,022 patients, incidence of delayed ejaculation was50 to 64% (48). Paroxetine, when used in doses of 10 to40 mg/day can increase IELT in 8,8 times comparing to baseline. Although with smaller impact on increasing ejaculatory time other SSRIs can be used in the following dosages: sertraline 50–200 mg, fluoxetine 20–40 mg and citalopram 20–40 mg. However, unlike Dapoxetine and its pharmokinetic profile, daily use of SSRIs is needed to maximize its therapeutic effect, which can be an advantage in couples with a high frequency of sexual encounters and interfere less in sexual spontaneity (39). On the other hand, side effects are increased (side effects include the ones described for dapoxetine and other sexual side effects as anejaculation, reduced libido and erectile dysfunction). SSRIs discontinuation syndrome represents a clinical entity with various psychological and neurovegetative symptoms that last for 3 to 4 days after sudden suspension of the drug which is followed by suicidal thoughts – these drugs require progressive and slow discontinuation before its suspension. From a financial point of view, SSRIs are cheaper compared to dapoxetine a fact that may allow for an increased frequency of sexual relations that with dapoxetine (49). *International Society for Sexual Medicine *guidelines determine that the off-label daily usage of paroxetine, sertraline, escitalopram and fluoxetine and on-demand treatment with paroxetine and sertraline for treatment of primary and acquired treatment of PE has an evidence level of 1a (30, 39).

***Tricyclic antidepressants (TCAs):*** Tricyclic antidepressants (TCAs) can also be used in an off-label regime. The most used drug for this end is clomipramine in the dosages of 10 to 50mg. By inhibiting the reuptake of catecholamines, it increases the adrenergic effects. It causes relevant cardiovascular side effects such as palpitations, hypotension and arrhythmia. Like SSRIs, clomipramine is recommended for off-label usage, as daily intake or on-demand by the ISSM (evidence level 1a) (30, 34, 39).

***Phospohodiesterase Type 5 Inhibitors (PDE5i):*** Phosphodiesterase Type 5 Inhibitors (PDE5i) are effective treatments used in erectile dysfunction (ED) and its use in premature ejaculation has been studied. Metanalyses conducted to date did not show solid evidence to support the use of PDE5i in PE, except in men with PE and DE. Recent studies suggest a possible role in the use of these drugs, as monotherapy, in patients with PE and normal erectile function (evidence level 4a according to ISSM guidelines, for off-label daily usage or on-demand in patients with PE and normal erectile function). PDE5i can be especially useful when there is ED and PE (30-50% of patients) (30).

***Central Acting Opioid Analgesics: ***Tramadol is a central acting opioid analgesic and studies demonstrate that it can increase IELT when given on demand or daily (34). Mechanism of action may be due to the antinociceptive secondary effects or relate to modulation of the nervous central system, reuptake of serotonin and norepinephrine (30).

Studies confirm that treatment with 25-100 mg of tramadol results in an increase of 2.4 to 12.6 times of basal IELT and can be dose dependant (34). It has a low safety profile, it presents addiction risk with chronic usage, it can cause dizziness, sleepiness, pruritus, dyspepsia, nausea, vomit and constipation. If combined with an SSRSI it can result in a Serotonin Syndrome, which can be potentially fatal, reason why this drug must be used with caution, and in selected patients, when other therapeutic approaches failed(evidence level 2 in accordance with ISSM guidelines) (30).

***Minimally invasive treatments: ***Acupuncture has demonstrated effectiveness in delaying ejaculation when compared to placebo, although being less effective than daily treatment with paroxetine (50).

Modulation and ablation of dorsal penile nerve (the main somatosensory pathway of the penis) or the increase of the penile gland by injection of hyaluronic acid have been suggested as useful treatments for PE (51, 52). Prologo et al. demonstrated that TC guided unilateral percutaneous cryoablation resulted in an significant increase in IELT in 24 men with treatment resistant PE (51).

Basal et al. evaluated the role of percutaneous pulse radiofrequency ablation of bilateral dorsal penile nerves in treating PE, observing that median IELT was significantly increased in 15 men with primary EP (52). 

***Future treatments:*** The main central targets identified include serotonergic, dopaminergic and oxytocinergic neurotransmitters, opioid receptors and mechanisms involved in the control of the spinal ejaculatory centre, located at T12-L1-L2. Peripheral interventions in the transport of ejaculatory content may also be able to delay ejaculation, diminishing sequential contraction of the epididymis, vas deferent, seminal vesicles, prostate and bladder neck. As such, a wide range of future treatment options is being researched for treatment of PE. Molecules such as DA-8031, silodosin, botulinum toxin-A and resiniferatoxin may be future treatment options for this disorder (53). The potential treatment options are summarized in table 5.

***Follow-up*** **: **Patients diagnosed with PE should be revaluated with the objective of: confirming the diagnosis; identify related comorbidities that may have not been identified previously; observe the patients evolution under treatment and couples satisfaction; confirm that treatment is being correctly taken or applied; monitor possible side effects and efficiency; adjust or if needed change treatment ;educate patients and recall recommendations and strategies (54).

## Conclusion

Although PE is the most frequent sexual dysfunction, it is still sub-diagnosed. An accurate clinical history is the best diagnostic method, and in the majority of the cases it is enough to differentiate between primary and acquired PE. Nowadays the treatment is not curative but is effective in increasing the IELT, improving the couple’s sexual satisfaction. Combining behavioural techniques with pharmacotherapy is the best way of treatment.
